# Mainstream smoke constituents and *in vitro* toxicity comparative analysis of 3R4F and 1R6F reference cigarettes

**DOI:** 10.1016/j.toxrep.2019.02.009

**Published:** 2019-02-25

**Authors:** Guy Jaccard, Donatien Tafin Djoko, Alexandra Korneliou, Regina Stabbert, Maxim Belushkin, Marco Esposito

**Affiliations:** PMI R&D, Philip Morris Products S.A., Quai Jeanrenaud 3, CH-2000, Neuchâtel, Switzerland

**Keywords:** Cigarettes mainstream smoke, Reference cigarettes, Cigarettes in vitro toxicity

## Abstract

A new Kentucky reference cigarette, 1R6F, has been manufactured to replace the depleting 3R4F reference cigarette. The 3R4F Kentucky reference cigarettes have been widely used as monitor or comparator cigarettes for mainstream smoke analysis and *in vitro* and *in vivo* toxicological data of cigarettes and novel tobacco products. Both reference cigarettes were analyzed in the same laboratory during the same period of time with the goal of performing a comparison of 3R4F and 1R6F. On the basis of the results obtained from aerosol chemistry and *in vitro* assays, we consider that the 1R6F reference cigarette is a suitable replacement for the 3R4F reference cigarette as a comparator/monitor cigarette. Its specific use as a comparator for novel tobacco products was checked on the basis of a comparative test with the Tobacco Heating System 2.2 as an example.

## Highlights

• 3R4F Kentucky reference cigarettes stock is depleting.
• 3R4F reference cigarettes have been widely used as monitor or comparator.
• 1R6F reference cigarettes are a suitable replacement for 3R4F on the basis of smoke chemistry and in vitro assays.

## Graphical abstract

**Figure:**
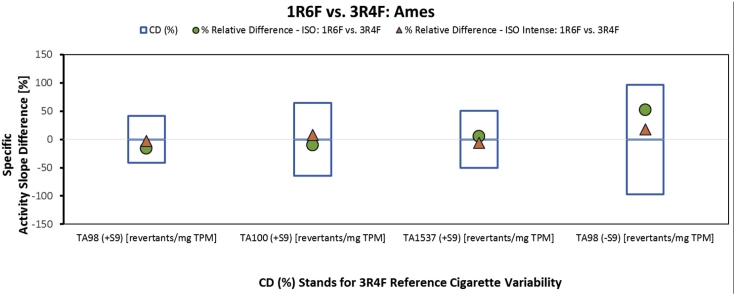

## 1 Introduction

The availability of reference cigarettes, prepared with a minimum of cigarette to cigarette variability and in quantities sufficient to cover a long period of time, is critical for laboratories performing smoke chemistry, *in vitro* testing, or *in vivo* analyses for cigarettes, novel tobacco products, and e-cigarettes, because reference cigarettes allow for the replication and the comparison over time of the test results obtained in other laboratories. In addition, reference cigarettes provide the most direct link between results obtained in each of the different pre-clinical trials mentioned above. Historically, the University of Kentucky provided such reference cigarettes, differing in their design and specifications, with the aim to represent various segments of the U.S. cigarette market. Lately, the Kentucky reference cigarette 3R4F has been widely used as a monitor or a comparator cigarette for mainstream smoke (MS) analyses and *in vitro* and *in vivo* toxicological assays. It has been regularly used as a monitor in collaborative tests organized for the analysis of MS constituents [[1], [2], [3]], or in the frame of methods development [[4], [5], [6], [7], [8], [9]], as a comparator for analyses performed for cigarettes [4,[10], [11], [12], [13], [14], [15]] or heated tobacco products [[16], [17], [18], [19], [20], [21], [22], [23]]. It has also been used as a comparator for toxicological *in vitro* assays for cigarettes [[24], [25], [26], [27]] or novel tobacco products and e-cigarettes [20,26,[28], [29], [30], [31], [32], [33], [34], [35], [36], [37], [38]] and *in vivo* toxicological studies [[39], [40], [41], [42], [43], [44], [45], [46]]. The stock for 3R4F reference cigarette is, however, depleting. Therefore, and upon service agreement with the U.S. Food and Drug Administration, the University of Kentucky produced 50 million cigarettes of a new Kentucky reference cigarette, 1R6F, in 2015, according to the specifications provided by the University of Kentucky (e.g., cigarette dimensions, amount of tobacco per cigarette, resistance to draw) [47]. Table 1 provides a comparison of the main cigarette design parameters of both 3R4F and 1R6F reference cigarettes.

**Table 1:** Main cigarette design parameters of 3R4F and 1R6F Kentucky reference cigarettes.

Parameter	3R4F (2006)	1R6F (2015)
*Blend (%)*		
Flue Cured	35.4	34
Burley	21.6	24
Maryland	1.4	–
Oriental	12.1	12
Reconstituted	29.6	20
Expanded Flue Cured		7
Expanded Burley	–	3
*Cigarette Design*		
Cigarette Length	84 mm	83 mm
Tobacco Rod Circumference	24.8 mm	24.6 mm
Tobacco Rod Length	57 mm	56 mm
Resistance to Draw	128 mm H2O	107
*Humectants (%)*		
Glycerol	2.7	1.7
Propylene glycol	–	1%
Isosweet	6.4	6.3%
*Cigarette Paper*		
Banded (CORESTA units)	–	9
Base (CORESTA units)	24	46
*Filter Ventilation (%)*	29	33
*Yield data from supplier*		
Puff count	9.0	7.5
TPM (mg/cig)	11.0	10.0
‘Tar’ (mg/cig)	9.4	8.6
Nicotine (mg/cig)	0.7	0.7
Carbon monoxide (mg/cig)	12.0	10.1

The 1R6F Kentucky reference cigarette has been used as a monitor or a comparator cigarette until now only in a limited number of cases, such as a comparator to heated tobacco products and e-cigarettes in *in vitro* or aerosol composition studies [19,48,49], in a smoking topography study [50], or for small cigar and cigarette studies [51,52]. It has also been used as a reference in a recently published recommended method of CORESTA [53].

The goal of the present study is to compare the MS chemistry and the *in vitro* cytotoxicity mutagenicity, and genotoxicity using standard assays, of the two Kentucky reference cigarettes, 3R4F and 1R6F. Such a comparison is necessary to determine whether both reference cigarettes are interchangeable or sufficiently similar to consider previous conclusions from a range of scientific studies performed with the 3R4F reference cigarette to be equally valid considering the chemistry and toxicity of the 1R6F reference cigarette. Such a study was performed in the past for the 2R4F (predecessor of 3R4F) and 3R4F reference cigarettes, and it was suggested that they were equivalent in terms of smoke chemistry and *in vitro* and *in vivo* toxicity [39].

## 2 Methods

### 2.1 MS analyses

The analyses were performed under Good Laboratory Practices at Labstat International ULC. The cigarette MS was generated under ISO [54] and ISO Intense [55] analytical smoking machine conditions. The cigarettes were conditioned before analysis according to standardized conditions [56].

The list of compounds analyzed in the MS and the related methods correspond to what was applied to the 3R4F cigarette in an already published article [20]. Four replicates per analysis were performed. The list covers common lists of harmful and potentially harmful constituents (HPHC), such as the World Health Organization-39 list [57] or the Health Canada list required for the reporting of commercial brands in Canada [58].

### 2.2 *In vitro* assay analyses

The mainstream cigarette smoke total particulate matter (TPM) was generated under ISO [54] and ISO Intense [55] analytical smoking machine conditions and extracted to a stock concentration of 10 mg/mL in dimethylsulfoxide. In addition, the mainstream gas vapor phase (GVP) was generated and tested in the neutral red uptake (NRU) assay only. The Health Canada methods T501, T502, and T503 [58] were applied to the Ames assay, NRU assay, and *in vitro* micronucleus (ivMN) assay, respectively. The vehicle and positive controls in each assay and on each day of testing were within the historical control ranges used in this laboratory; therefore, the assays were considered valid.

### 2.3 Statistical treatment of data

The overall rationale for the comparative assessment between the 1R6F and 3R4F Kentucky reference cigarettes is based on the fundamental notion of long-term analytical variability. In the context of a single batch of reference monitor test pieces manufactured at a single point in time, the long-term analytical variability [12] represents the natural variation from analytical results obtained in different studies conducted within the same laboratory, using the same methods but at different points in time. For each constituent and specified analytical smoking machine condition, the long-term analytical variability was empirically estimated through one year of measurements conducted on the 3R4F reference cigarette at Labstat International ULC. The magnitude of the difference between 3R4F and 1R6F is evaluated constituent by constituent against the corresponding inherent precision of the analytical method over time, which is essentially driven by the long-term variability for each constituent.

For each of the constituents above the limit of quantification (LOQ) in the 3R4F monitor test piece reference data set, the critical differences for differences of means of two single point in time measurements between 3R4F and 1R6F are defined to be [59]:

$$Critical\;difference,\;CD\left[\text{\%}\right]=\;3\text{*}\sqrt{2}\text{*}RSD\left[\text{\%}\right]\text{*}\sqrt{\frac{1}{n}}$$

where RSD[%] stands for the relative standard deviation in percent of the 3R4F monitor data set for the given constituent, and n = number of replicates (i.e., four replicates, as per the sample testing scheme). The performance of the 3R4F and 1R6F reference cigarettes are deemed comparable if the absolute percentage differences per constituent are within the performance boundaries expressed by the critical differences.

For endpoints where some replicates are below LOQ in the 3R4F monitor reference data set, the average yields are compared to a threshold established as 4*LOQ.

## 3 Results

### 3.1 MS chemistry

The relative percent differences for the mean individual HPHC deliveries of 1R6F cigarettes to 3R4F cigarettes were compared to the long-term 3R4F deliveries variability, according to a previously described method [12,60].

**Fig. 1:**
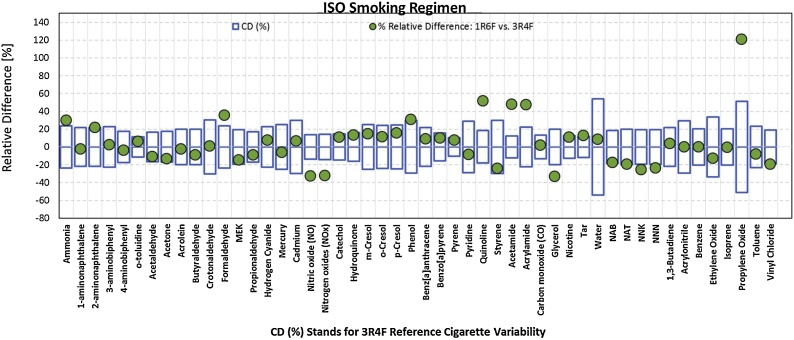
Graphical representation of the percentage difference between the yields of measured constituents in MS obtained under the ISO smoking regime from 1R6F and 3R4F reference cigarettes.

A graphical representation of the differences observed between 1R6F and 3R4F cigarettes is provided in Fig. 1.

Compounds not included in the critical difference statistical analysis, due to non-quantifiable results, include arsenic, chromium, lead, nickel, selenium, nitrobenzene, and resorcinol.

Statistically significant increases were observed for ammonia, formaldehyde, phenol, quinoline, acetamide, acrylamide, and propylene oxide under the ISO smoking regime and for propylene oxide using the ISO Intense smoking regime for 1R6F cigarettes.

**Fig. 2:**
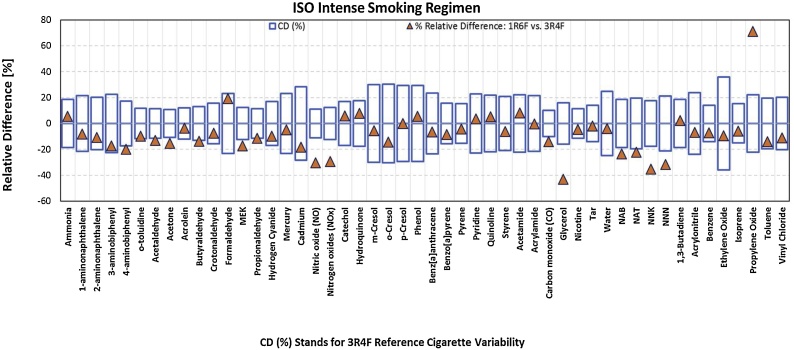
Graphical representation of the percentage difference between the yields of measured constituents in MS obtained under the ISO Intense smoking regime from 1R6F and 3R4F reference cigarettes.

Statistically significant decreases were observed for NO, NOx, NNK, NNN, and vinyl chloride under the ISO smoking regime and for 4-aminobiphenyl, acetaldehyde, acetone, butyraldehyde, MEK, propionaldehyde, NO, NOx, CO, NAB, NAT, NNK, and NNN under the ISO Intense smoking regime for 1R6F cigarettes (Fig. 2).

### 3.2 *In vitro* analyses

The 3R4F results for all *in vitro* assays and treatment conditions were within the expected (historical) range of 3R4F assay responses when the TPM was generated under intense smoking conditions. No historical ranges for the *in vitro* assays were calculated for the 3R4F MS when generated under ISO smoking conditions due to a lack of sufficient data.

#### 3.2.1 *In vitro* bacterial mutagenicity

Following treatment with the 3R4F and 1R6F TPM, concentration-related and reproducible increases in revertants were observed in tester strains TA98, TA100, and TA1537 in the presence of S9 and in TA98 in the absence of S9 compared to solvent control when the cigarettes were smoked under ISO and ISO Intense smoking conditions. In the remaining strains and treatment conditions, no reproducible increase in revertants reproducible was observed. For these tester strains that have been proven to be responsive to TPM, the specific mutagenicity (Ames assay-specific activity slope) was determined. Then, the relative % difference in specific mutagenicity between the 1R6F and the 3R4F was compared to the 3R4F long-term variability.

**Fig. 3:**
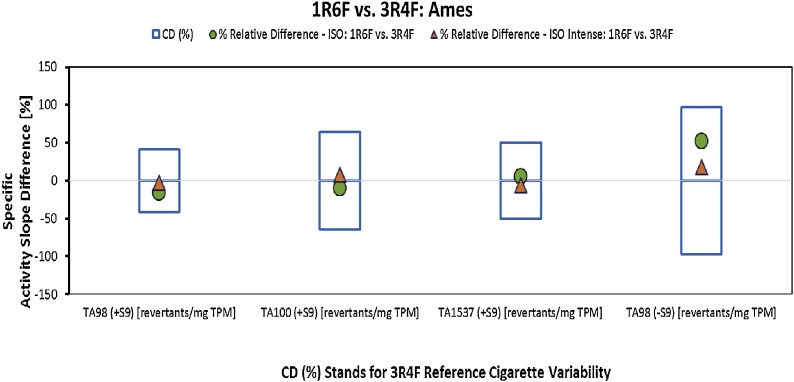
Relative % difference between Ames assay-specific activity slopes for TPM from MS of 1R6F and 3R4F cigarettes obtained under ISO (green circles) and ISO Intense (orange triangles) smoking conditions related to the long-term variability of the 3R4F (critical difference, CD).

This assessment showed that for the Ames assay, in any of the used strains in the presence and absence of S9 metabolic activation, the relative differences observed between the 1R6F- and 3R4F-specific mutagenicity did not exceed the calculated 3R4F long-term variability for the ISO or ISO Intense smoking regime, as shown in Table 3 and illustrated in Fig. 3.

#### 3.2.2 *In vitro* cytotoxicity (NRU assay)

**Fig. 4:**
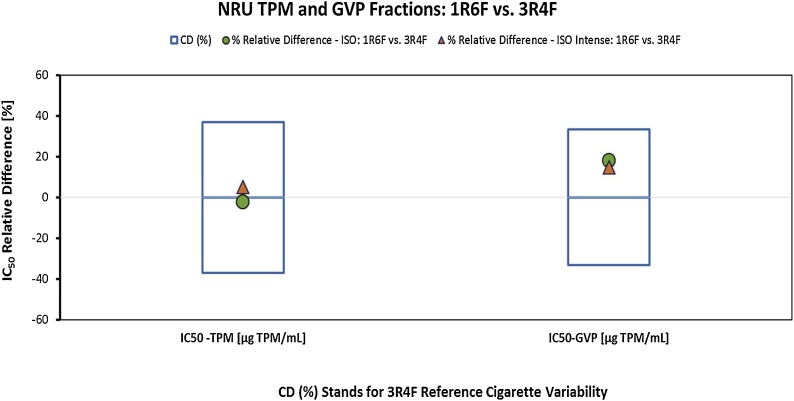
Relative % difference between IC_50_ for TPM and GVP from MS of 1R6F and 3R4F cigarettes obtained under ISO (green circles) and ISO Intense (orange triangles) smoking conditions related to the long-term variability of the 3R4F (critical difference, CD).

In the NRU assay, the TPM and GVP samples generated under ISO and ISO Intense smoking conditions induced concentration-related decreases in cell viability, and an IC_50_ value could be derived in each instance. The relative differences observed between the 1R6F and 3R4F response statistics for the NRU assay analysis did not exceed the calculated 3R4F reference item variability in any of the relevant smoking regimes or smoke fractions, as shown in Table 4 and illustrated in Fig. 4.

#### 3.2.3 *In vitro* genotoxicity (ivMN assay)

**Fig. 5:**
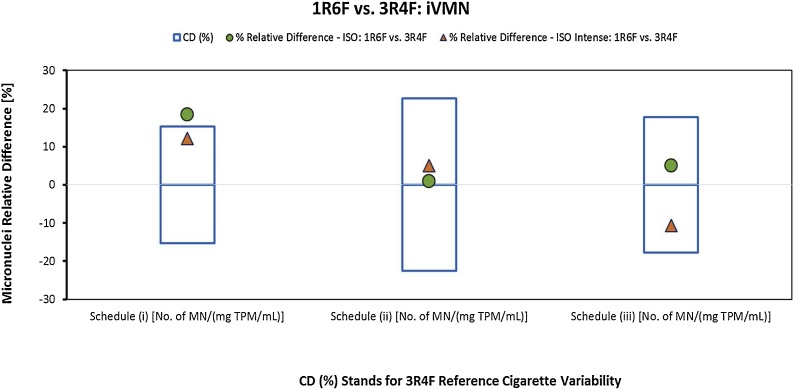
Relative % difference between ivMN linear regression slopes [number of MN/(mg TPM/mL)] for TPM from MS of 1R6F and 3R4F cigarettes obtained under ISO (green circles) and ISO Intense (orange triangles) smoking conditions compared to the long-term variability of the 3R4F (critical difference, CD) for each treatment schedule.

When the 1R6F and 3R4F TPMs were tested in the ivMN assay, the assay response exhibited an overall genotoxic response in each treatment condition (schedule (i), (ii), (iii)) and for each smoking regime. The schedules (i), (ii) and (iii) correspond to the schedules described in the Health Canada method T-503 [58] and correspond respectively to a short-term exposure of the cells in the absence of metabolic activation, a short-term exposure of the cells in the presence of metabolic activation and to a long-term exposure of the cells in the absence of metabolic activation. The relative difference observed between the 1R6F and 3R4F response statistics for the ivMN assay analysis did not exceed the calculated 3R4F long-term variability, with the exception of schedule (i) (short-term exposure in the absence of S9) when the TPM was generated under the ISO smoking regime, as shown in Table 5 and illustrated in Fig. 5.

### 3.3 Use of 3R4F or 1R6F as a comparator for heated tobacco products

The 3R4F cigarette has been used as a comparator for the aerosol composition of heated tobacco products [[17], [18], [19], [20],^22^,^26^], using different lists of HPHCs, with an average reduction of about 90% (e.g., for the Tobacco Heating System (THS) 2.2) across a broad range of chemical compounds in comparison with 3R4F [16]. It was also confirmed that the average reduction in aerosol yields for THS 2.2 when compared with 3R4F was equally valid for commercial cigarettes sampled worldwide [16].

**Fig. 6:**
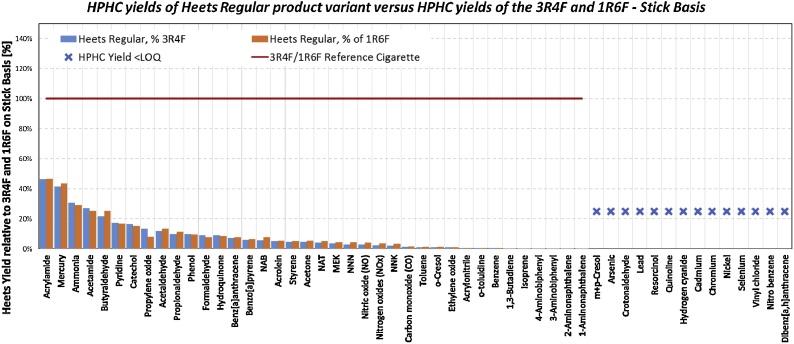
Reduction of HPHC yields for THS 2.2 in comparison with 3R4F (blue) and 1R6F (orange) cigarettes, using the ISO Intense analytical smoking machine regime.

The use of 1R6F and 3R4F cigarettes as comparators for the aerosol composition of a specific heated tobacco product, THS 2.2, is provided in the Fig. 6 below.

Using the results provided in Table 1 for 3R4F and 1R6F cigarettes and the results previously published for THS 2.2 [16], there is an average reduction of 93% and 92% across the Health Canada list of HPHCs for 3R4F and 1R6F cigarettes, respectively, or 91% across the full list provided in Table 2 in comparison with both 3R4F and 1R6F cigarettes.

**Table 2:** MS chemistry results for 3R4F and 1R6F reference cigarettes.

HPHC	3R4F ISO Mean	3R4F ISO SD	3R4F intense Mean	3R4F intense SD	1R6F ISO Mean	1R6F ISO SD	1R6F intense Mean	1R6F intense SD
Nicotine [mg/cig]	0,702	0,042	1,99	0,13	0,778	0,052	1,90	0,05
			*2,02*				*2,00*	*0,08*
CO [mg/cig]	10,0	0,6	30,5	1,8	10,2	0,4	26,2	0,6
			*32,0*	*1,0*			*29,4*	*0,6*
Ammonia [μg/cig]	9,64	1,06	34,0	0,5	12,5	0,5	35,9	1,7
			*32,5*	*3,5*			*34,7*	*2,0*
Formaldehyde [μg/cig]	24,7	2,4	87,1	7,4	33,5	3,7	104	9
			*54,1*	*6,0*			*68,4*	*3,9*
Acetaldehyde [μg/cig]	597	13	1838	105	532	9	1601	137
			*2200*	*103*			*1859*	*169*
Acetone [μg/cig]	241	7	749	44	209	7	635	57
			*660*	*24*			*520*	*42*
Acrolein [μg/cig]	53,2	1,4	180	13	51,8	2,1	173	17
			*159*	*9*			*148*	*22*
Propionaldehyde [μg/cig]	43,0	1,1	135	8	39,1	0,9	119	11
			*132*	*3*			*116*	*13*
Crotonaldehyde [μg/cig]	9,89	0,63	59,5	4,4	10,0	0,6	55,0	5,9
			*42,0*	*6,2*			*39,5*	*3,2*
Methyl Ethyl Ketone [μg/cig]	57,7	2,4	199	13	49,2	2,6	164	16
			*192*	*8*			*150*	*14*
Butyraldehyde [μg/cig]	28,4	0,7	93,0	4,3	25,9	1,0	80,3	7,7
			*60,9*	*5,1*			*51,5*	*7,3*
HCN [μg/cig]	91,5	8,3	390	23	98,3	14,0	352	10
			*343*	*62*			*332*	*43*
Mercury [ng/cig]	2,10	0,03	4,92	0,06	1,97	0,13	4,68	0,24
			*4,26*	*0.50*			*3,89*	*0,32*
Cadmium [ng/cig]	24,5	2,0	93,2	7,4	26,1	2,3	76,1	1,2
			*105*	*5*			*88,8*	*1,9*
Lead [ng/cig]	<LOQ	NA	<LOQ	NA	<LOQ	NA	<LOQ	NA
			*28,7*	*0,8*			*28,1*	*0,6*
Chromium [ng/cig]	<LOD	NA	<LOD	NA	<LOD	NA	<LOD	NA
			*<LOQ*	*NA*			*<LOQ*	*NA*
Nickel [ng/cig]	<LOD	NA	<LOD	NA	<LOD	NA	<LOD	NA
			*<LOQ*	*NA*			*<LOQ*	*NA*
Arsenic [ng/cig]	<LOQ	NA	<LOQ	NA	<LOQ	NA	<LOQ	NA
			*8,01*	*0.56*			*7,57*	*0,27*
Selenium [ng/cig]	<LOD	NA	<LOD	NA	<LOD	NA	<LOD	NA
			*<LOQ*	*NA*			*<LOQ*	*NA*
NO [μg/cig]	196	9	471	17	132	6	329	13
			*495*	*16*			*357*	*24*
NOx [μg/cig]	211	9	524	17	143	6	369	15
							*405*	*26*
			*555*	*19*				
Pyridine [μg/cig]	7,46	1,34	35,2	6,4	6,83	0,73	36,4	4,6
			*28,6*	*2,8*			*30,4*	*2,4*
Quinoline [μg/cig]	0,136	0,009	0,315	0,033	0,207	0,015	0,331	0,041
			*0,389*	*0,028*			*0,427*	*0,009*
Styrene [μg/cig]	6,54	1,17	21,7	2,4	4,97	0,67	20,4	2,3
							*14,8*	*0,9*
			*16,1*	*2,0*				
Nitrobenzene [μg/cig]	<LOD	NA	<LOD	NA	<LOD	NA	<LOD	NA
			*<LOD*	*NA*			*<LOD*	*NA*
Hydroquinone [μg/cig]	32,4	1,0	78,1	6,5	36,7	1,8	84,2	5,6
							*88,7*	*6,2*
			*84,2*	*1,8*				
Resorcinol [μg/cig]	<LOD	NA	<LOQ	NA	<LOQ	NA	<LOQ	NA
							*1,80*	*0,15*
			*1,57*	*0,22*				
Catechol [μg/cig]	39,5	1,5	86,9	5,4	43,8	1,7	91,9	5,7
			*87,4*	*3,4*			*91,8*	*5,3*
Phenol [μg/cig]	6,99	0,36	11,1	0,9	9,13	0,58	11,7	0,6
			*13,5*	*0,8*			*12,5*	*0,6*
p-Cresol [μg/cig]	4,47	0,22	6,83	0,53	5,16	0,21	6,84	0,53
			*8,72*	*0,38*				
							*7,77*	*0,41*
m-Cresol [μg/cig]	1,89	0,13	2,77	0,19	2,18	0,16	2,62	0,14
			*3,48*	*0,18*			*2,98*	*0,07*
o-Cresol [μg/cig]	2,22	0,25	3,22	0,27	2,47	0,08	2,76	0,22
			*3,94*	*0,16*			*3,12*	*0,13*
Pyrene	37,3	0,7	91,9	7,5	40,2	0,7	88,0	6,0
			*79,4*	*7,5*			*68,4*	*10,3*
Benzo(a) anthracene [ng/cig]	12,1	0,2	28,9	2,0	13,2	0,1	27,0	1,3
			*24,2*	*2,4*			*21,4*	*3,2*
Benzo(a)pyrene [ng/cig]	6,30	0,21	15,1	0,6	6,94	0,17	13,8	0,3
			*12,9*	*1,3*			*11,4*	*1,7*
Dibenz(a,h) anthracene [ng/cig]	<LOQ	NA	1,34	0,09	<LOQ	NA	1,19	0,20
			*0,915*	*0,124*			*0,892*	*0,086*
1,3-Butadiene [μg/cig]	37,9	3,1	100	7	39,3	3,0	102	5
							*114*	*4*
			*108*	*4*				
Isoprene [μg/cig]	288	18	799	61	286	24	752	36
			*887*	*49*			*859*	*46*
Acrylonitrile [μg/cig]	5,23	0,51	20,5	1,8	5,23	0,67	19,2	1,3
							*18,5*	*1,9*
			*19,5*	*1,6*				
Benzene [μg/cig]	33,6	2,6	88,8	6,3	33,7	2,8	82,3	4,6
			*78,6*	*4,6*			*76,0*	*5,8*
Toluene [μg/cig]	51,4	4,8	153	12	47,2	4,0	132	9
			*131*	*5*			*116*	*9*
Vinyl Chloride [ng/cig]	39,6	3,5	94,6	7,4	31,9	2,2	84,0	3,5
							*109*	*19*
			*95,6*	*9,2*				
Ethylene oxide [μg/cig]	6,78	0,32	19,2	1,8	5,92	0,63	17,3	0,8
			*19,3*	*2,0*			*17,2*	*0,9*
Propylene oxide [μg/cig]	297	54	1000	79	657	82	1710	138
			*903*	*308*			*1692*	*232*
1-Aminonaphthalene [ng/cig]	14,2	1,0	29,1	2,6	13,8	1,4	26,7	0,8
			*17,6*	*0,6*			*17,2*	*0,6*
2-Aminonapthalene [ng/cig]	8,77	1,11	18,1	2,3	10,7	0,8	16,2	1,3
			*13,2*	*0,8*			*11,8*	*0,9*
3-Aminobiphenyl [ng/cig]	2,27	0,09	5,44	0,23	2,33	0,28	4,49	0,39
			*3,49*	*0,27*			*3,07*	*0,25*
4-Aminobiphenyl [ng/cig]	1,54	0,12	3,91	0,29	1,48	0,05	3,13	0,04
							*1,91*	*0,23*
			*2,29*	*0,12*				
o-Toluidine [ng/cig]	55,9	6,9	121	1,0	59,4	4,9	109	1
							*84,6*	*2,2*
			*83,3*	*2,1*				
NNN [ng/cig]	131	8	337	28	100	9	230	17
			*263*	*12*			*191*	*8*
NAT [ng/cig]	136	4	334	12	110	10	259	30
							*246*	*12*
			*268*	*20*				
NAB [ng/cig]	13,3	0,6	31,8	1,0	11,0	1,1	24,4	2,2
			*24,1*	*1,1*			*21,3*	*1,6*
NNK [ng/cig]	113	8	295	32	84,5	6,0	192	8
			*281*	*16*			*208*	*7*
Acetamide [μg/cig]	2,70	0,18	12,2	1,9	3,99	0,42	13,2	1,0
			*11,9*	*1,0*			*14,0*	*1,0*
Acrylamide [μg/cig]	1,07	0,05	3,92	0,52	1,58	0,14	3,91	0,33
			*3,99*	*0,39*			*4,49*	*0,34*

Data in italics correspond to the results published by Forster et al [19]. NAB stands for N’-nitrosoanabasine, NAT for N’-nitrosoanatabine, NNN for N’-nitrosonornicotine and NNK for 4-(methylnitrosamino)-1-(3-pyridyl)-1-butanone.

**Table 3:** Comparison of relative % difference between mean 1R6F- and 3R4F-specific activities (revertants/mg TPM) to 3R4F long-term variability.

*Note*:  statistical analysis not done because at least one test item was determined to be non-mutagenic overall.

The relative (%) difference is compared to the calculated 3R4F long-term variability for the assay statistic to determine if the difference exceeds the long-term variability of the Ames test method.

**Table 4:** Comparison of % relative difference between mean IC_50_ (μg TPM/mL or μg TPM equivalent/mL) of 1R6F and 3R4F and the 3R4F long-term variability (significant differences of NRU assay).

					1R6F IC_50_		3R4F IC_50_		Mean IC_50_ Comparisons	
					[mg/mL]		[mg/mL]			
Smoking Regime^3^	Assay	Smoke Fraction^4^	Cigarette Comparison	3R4F Reference Item Cigarette Variability (%)	Average	Std. Dev.	Average	Std. Dev.	Observed Relative Diff. (%)	Observed Exceeds 3R4F Variability?
ISO	NRU	TPM	1R6F vs. 3R4F	37.1	68.4	10.4	69.9	13.2	−2.2	no
ISO	NRU	GVP	1R6F vs. 3R4F	33.3	160.5	39.6	136.0	17.2	18.0	no
Intense	NRU	TPM	1R6F vs. 3R4F	37.1	72.2	12.6	68.6	1.4	5.2	no
Intense	NRU	GVP	1R6F vs. 3R4F	33.3	149.5	10.0	130.2	14.6	14.9	no

*Note*: The relative (%) difference is compared to the calculated 3R4F long-term variability for the assay statistic to determine if the difference exceeds the long-term variability of the NRU test method.

**Table 5:** Comparison of relative % difference between 1R6F and 3R4F linear regression slopes [number of MN/(mg TPM/mL)] to 3R4F long-term variability for ivMN test.

						**Linear Regression Slope Comparisons**	
**Smoking Regime^2^**	**Schedule**	**Brand Comparison**	**3R4F Reference Item Cigarette Variability**	**1R6F Linear Regression Slope**	**3R4F Linear Regression Slope**	**Observed Relative Diff.**	**Observed Exceeds 3R4F Variability?**
			****(%)****	****(No. of MN/ (mg TPM/mL))****	****(No. of MN/ (mg TPM/mL))****	****(%)****	
ISO	Schedule (i)	1R6F vs. 3R4F	15.3	61.5	51.9	18.5	**yes**
ISO	Schedule (ii)	1R6F vs. 3R4F	22.6	30.1	29.9	0.9	no
ISO	Schedule (iii)	1R6F vs. 3R4F	17.7	63.9	60.9	5.0	no
Intense	Schedule (i)	1R6F vs. 3R4F	15.3	57.6	51.4	12.2	no
Intense	Schedule (ii)	1R6F vs. 3R4F	22.6	34.7	33.0	5.0	no
Intense	Schedule (iii)	1R6F vs. 3R4F	17.7	56.8	63.5	−10.6	no

Note: The relative (%) difference is compared to the calculated 3R4F long-term variability for the assay statistic to determine if the difference exceeds the long-term variability of the ivMN test method.

## 4 Discussion

Due to the depletion of 3R4F cigarette stock, there will soon be a need to use other reference cigarettes for comparative or monitoring purposes (e.g., for studies performed for cigarettes and novel tobacco products). The recently produced 1R6F Kentucky reference cigarettes are an obvious candidate to replace the 3R4F Kentucky reference cigarettes. We performed a study that included smoke chemistry and *in vitro* toxicological assays and used both ISO and ISO Intense smoking regimes, analogous to what was performed in the past to compare the 2R4F (predecessor of 3R4F) and 3R4F reference cigarettes [39].

The MS chemistry of both reference cigarettes, according to the Health Canada list of HPHCs or the wider PMI list [20] obtained under ISO and ISO Intense analytical smoking regimes, has been measured. There are some HPHCs that are either significantly higher or significantly lower in the MS of 1R6F cigarettes when compared with 3R4F cigarettes. It should be noted that with the statistical approach taken in our work [12], one expects to have 0.05*N (where N is the number of HPHC = 58), or about three occurrences of a single HPHC being significantly different when comparing 1R6F and 3R4F. Additionally, it is not expected to have the exact same profile of HPHCs in both reference cigarettes, because the cigarette design is not exactly the same, both in terms of blend (with tobaccos grown in different conditions in terms of soils, locations and environment) and cigarette construction, according to the specifications provided by the University of Kentucky (e.g., cigarette dimensions, filter properties, etc.).

Some HPHCs, such as NO, NOx, NNN, and NNK, are significantly lower in the 1R6F cigarettes than in 3R4F cigarettes with both analytical smoking regimes. Those compounds may all be affected by the blend composition [61]; NO content in smoke is primarily determined by the nitrate content in the tobacco blend [61], and NNN and NNK are typically higher in air-cured tobaccos than in flue-cured tobaccos [62], with a tendency for a downward trend in recent years due to the introduction of new agricultural and curing practices [63,64]. The lower NNN content observed in the MS of 1R6F cigarettes is also observed in the tobacco blend, with reported values for NNN in 1R6F of 2,131 ng/g [47] or 2,294 ng/g [65] and in 3R4F of 2,636 ng/g [66]. However, this is not the case for NNK, for which the reported values are very similar, with values in 1R6F of 676 ng/g [47] and 675 ng/g [65] and in 3R4F of 679 ng/g [66]. The lower value observed for 1R6F MS NNK yields may be due to a lower transfer from tobacco to smoke of NNK, related to a lower amount of bound NNK in the blend [67,68].

Some HPHCs are significantly lower in 1R6F only for one of the two smoking regimes, such as NAB, NAT, vinyl chloride, some aldehyde compounds, CO, or 4-aminobiphenyl. This difference can be due either to the statistical approach mentioned above or to changes in the blend design (e.g., for NAB, NAT, or 4-aminobiphenyl) and/or cigarette design.

One compound, propylene oxide, is significantly higher in 1R6F for both analytical smoking regimes. A possible source for propylene oxide is propylene glycol used as humectant in tobacco blends [69,70]. There is a difference of 1% propylene glycol in the recipes of 1R6F and 3R4F cigarettes (1% in 1R6F; 0% in 3R4F). Gaworski et al. [70] observed an increase of propylene oxide with increasing amounts of added propylene glycol. On the basis of their results, an increase of about 850 ng/cigarette of propylene oxide using the ISO smoking regime could be expected for an addition of 1% propylene glycol, for cigarettes differing only in their amount of added propylene glycol. We observe an increase of 360 ng/cigarette between the 1R6F and the 3R4F using ISO smoking regime, consistent with Gaworski et al. observation, noting that the design of 3R4F and 1R6F cigarettes is not the same.

Finally, some compounds are higher in 1R6F only for one of the two smoking regimes. The remarks provided above for the compounds that are higher in 3R4F cigarette MS are valid in this case, as well.

In terms of *in vitro* assay results, 1R6F and 3R4F cigarettes displayed similar *in vitro* cytotoxicity, mutagenicity, and genotoxicity under both smoking conditions, with the exception of a statistically significantly higher response of the 1R6F in the ivMN assay (schedule (i)) under ISO smoking conditions. Considering the smoke chemistry results for the 1R6F and the 3R4F, the increase in ivMN (schedule (i)) may be explained by the increase in acetamide and acrylamide in MS from the 1R6F. Although propylene oxide and formaldehyde were also increased in 1R6F MS, both constituents are mainly in the GVP, and their increase cannot explain the increase in genotoxicity of the 1R6F TPM extract. Most of the carcinogens in TPM (e.g., NNK, NNN, cadmium) were lower in 1R6F TPM or were not different between the cigarettes. Acetamide, which is classified as 2B by the International Agency for Research on Cancer (IARC) [71], is a TPM constituent and was increased by 48% under ISO smoking conditions. Acrylamide, which is classified as 2A by IARC, is also a TPM constituent and was also increased by 48% under ISO smoking conditions.

In the literature, acrylamide did not increase the mutation frequency in bacteria (e.g., TA98 and TA100). Furthermore, there is consistent evidence that activity of acrylamide in cultured mammalian cells was also seen in the absence of an exogenous metabolic activation system, implying that metabolic activation to its metabolite glycidamide might not be necessary to present its genotoxic properties [72]. All *in vivo* MN assays in mouse bone marrow cells, for example, also showed positive results without exogenous metabolic activation [72].

The toxicological profile of acetamide is less clear. In the literature, acetamide was not mutagenic in the Ames assay [71]. It was marginally positive in the induction of bone marrow micronuclei in male C57BL/6 mice in one study, but it was negative in another study at higher doses in the same species as well as in CBA male mice [71].

In summary, the physicochemical and toxicological profiles of acetamide and acrylamide are capable of explaining the increase in ivMN seen for 1R6F TPM; hence, the two smoke constituents may contribute to this genotoxic effect. However, the question arises why the results of schedule (iii) did not support the results of schedule (i). Furthermore, the 3R4F long-term variability of 15% for the ivMN (schedule (i)) seems to be very low compared with the long-term variabilities seen for the Ames assay and the NRU assay. Therefore, the increase in genotoxicity for 1R6F TPM compared with 3R4F TPM may be a chance finding. Future genotoxicity studies with 1R6F MS will show if the increase in ivMN (schedule (i)) compared with 3R4F MS is reproducible and meaningful.

When 3R4F and 1R6F cigarettes were used as comparison cigarettes to evaluate the aerosol composition of a heated tobacco product, THS 2.2, the average reduction was almost identical with both reference cigarettes. This was also true when comparing the aerosol composition of THS 2.2 with a range of commercially available cigarettes or with 3R4F reference cigarettes [16].

In conclusion, there are some slight differences in terms of smoke chemistry and *in vitro* assays for the MS of 1R6F and 3R4F reference cigarettes, obtained under ISO and ISO Intense analytical smoking machine regimes. Those differences did not, however, translate into different conclusions regarding the reduction of HPHCs in a heated tobacco product, THS 2.2.

## Transparency document

Transparency document
